# Novel role for the Golgi membrane protein TMEM165 in control of migration and invasion for breast carcinoma

**DOI:** 10.18632/oncotarget.27668

**Published:** 2020-07-14

**Authors:** Pavitra Murali, Blake P. Johnson, Zhongpeng Lu, Leslie Climer, Danielle A. Scott, Francois Foulquier, Gabriela Oprea-Ilies, Vladimir Lupashin, Richard R. Drake, Karen L. Abbott

**Affiliations:** ^1^University of Oklahoma Health Sciences Center, Department of Biochemistry and Molecular Biology, Oklahoma City, OK, United States; ^2^Ouchita Baptist University, Department of Biology, Arkadelphia, AR, United States; ^3^Centre National de la Recherche Scientifique, Unité Mixte de Recherche, Unité de Glycobiologie Structurale et Fonctionnelle, University of Lille, Lille, France; ^4^University of Arkansas for Medical Sciences, Department of Biophysics and Physiology, Little Rock, AR, United States; ^5^Emory University, Pathology and Laboratory Medicine, Atlanta, GA, United States; ^6^Department of Cell and Molecular Pharmacology, Medical University of South Carolina, Charleston, SC, United States

**Keywords:** TMEM165, migration, invasion, breast cancer, glycosylation

## Abstract

The *TMEM165* gene encodes for a multiple pass membrane protein localized in the Golgi that has been linked to congenital disorders of glycosylation. The TMEM165 protein is a putative ion transporter that regulates H^+^/Ca^++^/Mn^++^ homeostasis and pH in the Golgi. Previously, we identified TMEM165 as a potential biomarker for breast carcinoma in a glycoproteomic study using late stage invasive ductal carcinoma tissues with patient- matched adjacent normal tissues. The TMEM165 protein was not detected in non-malignant matched breast tissues and was detected in invasive ductal breast carcinoma tissues by mass spectrometry. Our hypothesis is that the TMEM165 protein confers a growth advantage to breast cancer. In this preliminary study we have investigated the expression of TMEM165 in earlier stage invasive ductal carcinoma and ductal carcinoma *in situ* cases. We created a CRISPR/Cas9 knockout of TMEM165 in the human invasive breast cancer cell line MDAMB231. Our results indicate that removal of TMEM165 in these cells results in a significant reduction of cell migration, tumor growth, and tumor vascularization *in vivo*. Furthermore, we find that TMEM165 expression alters the glycosylation of breast cancer cells and these changes promote the invasion and growth of breast cancer by altering the expression levels of key glycoproteins involved in regulation of the epithelial to mesenchymal transition such as E-cadherin. These studies illustrate new potential functions for this Golgi membrane protein in the control of breast cancer growth and invasion.

## INTRODUCTION

Breast cancer is the most commonly diagnosed cancer in women. It is estimated that 1 in 8 women will be diagnosed with the breast cancer in the United States leading to over 252,710 new cases of breast cancer each year. More than 40,500 women will die of breast cancer each year [1]. Increased rates of mammography screening have led to an increase in the diagnosis of ductal carcinoma *in situ* (DCIS), an abnormal proliferation of epithelial cells in the breast ducts that has not invaded tissue and is not cancer. While DCIS is considered a precursor to invasive ductal carcinoma (IDC) in certain cases; only 20–50% of DCIS cases will progress to IDC [2–5]. Currently, there are no efficient diagnostic methods to distinguish DCIS cases that will remain indolent from those that will progress to IDC. The discovery of molecular markers that could identify DCIS cases with a higher risk of progression to invasive cancer would be a significant clinical advance. Studies have revealed that many factors including altered patterns of gene expression and post-translational regulation contribute to the progression of DCIS to IDC [6–9]. Studies focusing on the characterization of molecular changes in early disease that will contribute to the conversion to invasive disease may lead to biomarkers useful for identifying which cases of DCIS may progress. TMEM165, a Golgi membrane protein, was discovered as a potential biomarker for invasive ductal breast carcinoma in our previous glycoproteomic study [10]. The TMEM165 protein was identified by mass spectrometry in invasive breast carcinoma tissue with no detection in patient-matched adjacent normal breast tissues. *TMEM165* is a gene found to be a putative ion transporter mutated in patients with congenital disorders of glycosylation [11–14]. CDGs are an increasing group of genetic metabolic disorders that affect protein glycosylation [15]. CDG patients with TMEM165 mutations were characterized by multiple system defects and in particular growth retardation due to bone and cartilage defects [13, 14]. A TMEM165-deficient zebrafish model exhibited phenotypic patterns such as bone dysplasia and abnormal cartilage development similar to the major clinical findings found in the three patients with a homozygous splice mutation [16]. Recently, CRISPR-Cas9 mediated genome wide screening in bacterial toxins revealed that TMEM165 as a critical Golgi protein required for maintaining proper levels of glycosylation [17].

**Figure 1:**
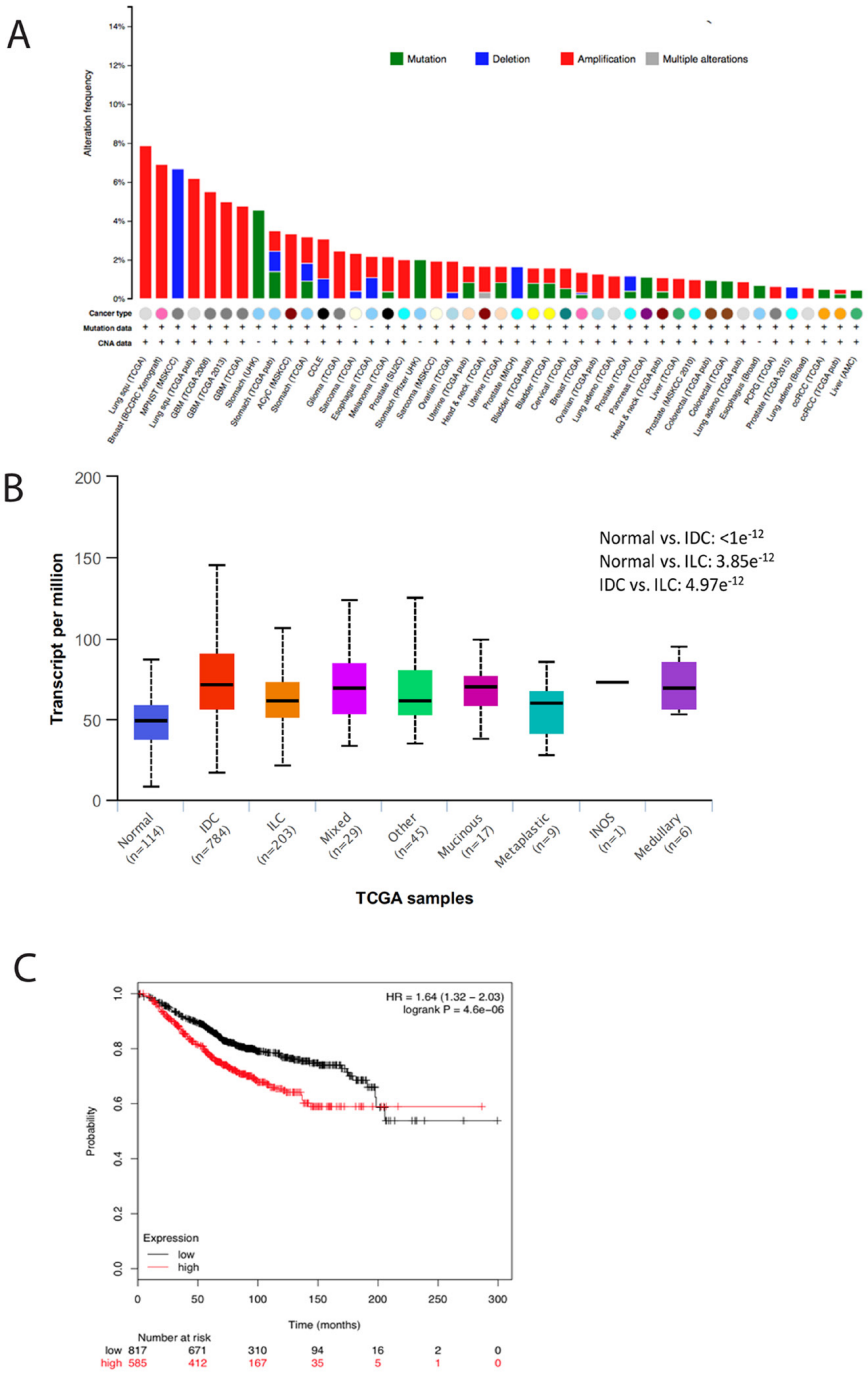
TMEM165 is increased in many human cancers and correlates with reduced overall survival. (**A**) Amplification of TMEM165 in human cancers in the cBioPortal [58, 59]. (**B**) Analysis of TMEM165 expression levels in molecular subtypes of human breast cancer using UALCAN. (**C**) Kaplan–Meier analysis (http://kmplot.com/analysis/index) of OS was plotted for breast cancer patients (*n* = 626). The OS was determined to be significantly longer in the *TMEM165* low expression group than in the *TMEM165* high expression group. A cutoff value of 1495 was chosen by auto select in the analysis configuration, with the expression value of the probe (218095_s_at) ranging from 89 to 8312. Upper quartile survival rates (months) for the low and high expression groups were 143 and 68.4, respectively.

The expression of TMEM165 is amplified in several human cancers (Figure 1A). We have analyzed TCGA breast cancer cases to examine TMEM165 expression levels in all molecular types of human breast cancer using UALCAN [18] (Figure 1B). We find that TMEM165 is amplified across all types of breast cancer compared to normal breast tissue with IDC cases having the highest levels of TMEM165 expression. The role of TMEM165 in normal breast physiology has been examined in lactating breast tissue. TMEM165 expression was upregulated during lactation 25 times and downregulated 95 times in involution [19]. TMEM165 has been demonstrated to maintain Ca++ and Mn++ ion homeostasis to support proper lactose synthetase functions during milk production in lactating breast tissues [20].

In the present study, we report that TMEM165 is upregulated in human breast cancer cell lines and patient tumor tissues and increased expression of TMEM165 correlates with poor prognosis in breast cancer patients. Using a CRISPR/Cas9 mediated TMEM165 knockout in the human breast cancer cell line MDAMB231 we find that TMEM165 deletion impaired invasive and migratory properties *in vitro* and tumor growth *in vivo*. We observed an inverse correlation between the protein expression levels of TMEM165 and E cadherin in several human breast cancer cell lines. Our TMEM165KO xenograft tumors show a re-expression of E-cadherin. E-cadherin is a well-known epithelial marker important for maintaining cell-cell adherens junctions which are crucial for tissue integrity and control of epithelial-to-mesenchymal (EMT) transitions during normal development. These findings place TMEM165 as a potential regulator controlling the EMT transition [21, 22]. Mechanistically, we found that TMEM165 expression induced changes in N-linked glycosylation. Our analysis of xenograft tissues reveals distinct changes in N-linked glycosylation as a result of TMEM165KO. Thus, we postulate that TMEM165 plays a key role in modulating breast cancer invasion by altering N-linked glycosylation leading to the suppression of E-cadherin levels. Collectively, our data demonstrate that overexpression of TMEM165 promotes EMT in breast cancer suggesting a novel role for TMEM165 as a driver of tumor invasion making it a prognostic marker and potential therapeutic target for breast cancer.

## RESULTS

### TMEM165 expression is increased in invasive breast cancer

**Figure 2:**
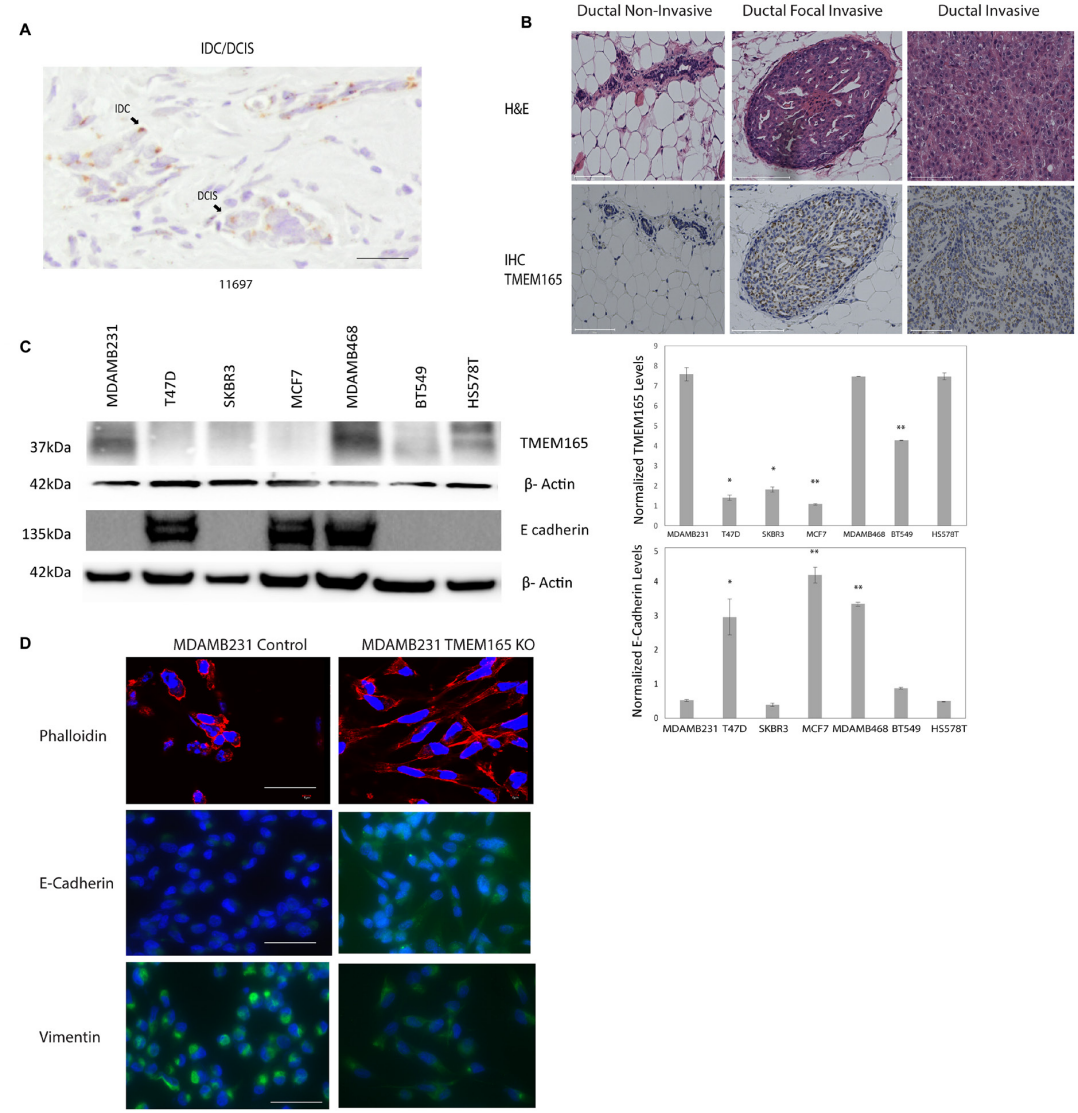
TMEM165 is increased in human breast cancer tissues and cell lines. (**A**) Immunohistochemistry staining for TMEM165 in IDC/DCIS breast cancer, arrows mark regions of IDC and DCIS, Bar = 100 μm. (**B**) Hematoxylin and eosin staining (top) and immunohistochemistry staining for TMEM165 (bottom) for MCF10DCIS xenograft tissues non-invasive, focal invasive, and invasive, Bar = 100 μm. (**C**) Western blot analysis showing TMEM165 and E cadherin expression levels across different human breast cancer cell lines. Right panel shows the quantification of TMEM165 and E-cadherin protein levels after normalization with actin (*n* = 2, ^*^ *P* < 0.05, ^**^ *P* < 0.01) (**D**) Phalloidin staining (top panel) to visualize actin filaments of MDAMB231 Control and MDAMB231 TMEM165KO cells seeded on fibronectin coated coverslips, Bar = 100 μm. Representative images of MDAMB231 Control and MDAMB231 TMEM165KO cells seeded on fibronectin coated coverslips stained for Vimentin and E-Cadherin with DAPI stained nuclei. Scale bar, 75 μm.

We have previously identified TMEM165 as a potential biomarker in a glycoproteomic analysis of high grade IDC cases with patient-matched adjacent normal tissues [10]. The cases that were analyzed in this study were all stage III or IV IDC. In the current study we were interested in determining the expression levels of TMEM165 in early stage (stage 1) IDC and DCIS cases. We conducted an initial analysis of a small number of DCIS or DCIS cases with regions of IDC to determine the TMEM165 levels in early IDC (Table 1, Figure 2A representative IHC). Over 95 positive cells were scored in each section and the representative case shown in Figure 2A had an overall intensity score of 8 for IDC with an overall intensity score of 5 for the DCIS section for the same patient. All IHC staining results (Table 1) show that in cases with both DCIS and IDC the TMEM165 levels increased in IDC in the same tissue section. We have further investigated the expression of TMEM165 in early invasive disease using the MCF10DCIS xenograft model. The MCF10DCIS cell line slowly progresses from DCIS to invasive lesions in 10 weeks. Data shown in Figure 2B demonstrate that TMEM165 is not expressed in the non-invasive stage, increases in the focal invasive stage while still contained within the basement membrane and continues to expand expression in the invasive stage. Next we looked at expression levels for TMEM165 in the TCGA breast carcinoma database [23]. The expression profile of TMEM165 among the histological subtypes of invasive breast cancer indicate that TMEM165 expression occurs in all subtypes with the highest percentages in the ER negative subtypes (Table 2, IDC subtypes). The substantial number of cases with increased TMEM165 expression led us to evaluate the prognostic significance of TMEM165 expression in breast cancer using the TCGA data. We find a highly significant reduction in overall survival in cases with higher TMEM165 expression (Figure 1C). We have analyzed TMEM165 expression across a panel of human breast cancer cell lines. TMEM165 expression levels were detected by Western blot in ER negative subtypes such as: MDAMB231 [24], BT549 [25], and HS578T [26] with no detection in the ER positive subtypesT47D, and MCF7 [27, 28] (Figure 2C). Real-time PCR analysis was performed on all cell lines and there was no TMEM165 mRNA detected in MCF7, and T47D cell lines (data not shown). Overall these results suggest that cell lines that express the ER receptor have very low or undetectable levels of TMEM165 protein expression. We observed an inverse trend between TMEM165 and E- cadherin expression levels in certain cell lines (Figure 2C). Cell lines exhibiting less invasiveness (MCF7 and T47D) expressed higher E-cadherin and non-detectable TMEM165. Whereas cell lines with high invasiveness (MDAMB231, BT549, and HS578T) expressed TMEM165 protein with lower E-cadherin expression (Figure 2C).

**Table 1:** Immunohistochemistry staining for TMEM165 in DCIS and early IDC cases

Cases	Stain Result	%cells TMEM165	Location	Intensity Score^a^
20659 DCIS/IDC	Pos	> 95	Perinuclear	IDC > DCIS (10 > 8)
13646 DCIS	Pos	> 95	Perinuclear	8
11697 DCIS/IDC	Pos	> 95	Perinuclear	IDC > DCIS (8 > 5)

^a^Percent positive cells were scored by a Pathologist and the overall intensity score is the mean intensity for all cells analyzed.

**Table 2:** TMEM165 expression in IDC vary by molecular subtypes

Subtype	Hormonal Status	Percentage of Breast invasive carcinoma cases showing alterations in TMEM165 gene expression^a^
Luminal A	ER+ and/or PR+, HER2, low Ki67	2%
Luminal B	ER+ and/or PR+, HER2 (or HER2- with high Ki67)	9%
Triple negative/Basal like	ER-, PR-, HER2-	15%
Her 2	ER-, PR-, HER2+	27%

^a^Database used was The Cancer Genome Atlas (TCGA), Nature (2012).

A CRISPR/Cas9 TMEM165 knockout (KO) was generated using the Claudin-low human breast cancer cell line MDAMB231 with guide RNAs (gsRNA) to TMEM165 previously described [29]. We have validated TMEM165KO cells by Western blot and immunohistochemistry as shown in Supplementary Figure 1. TMEM165KO or control cells were stained with the toxin phalloidin to visualize actin structures [30]. TMEM165KO cells displayed a significant change in morphology with reorganization of actin and increased cell spreading on fibronectin (Figure 2D). TMEM165KO cells also had elevated levels of E-cadherin compared with control cells. The levels of vimentin, a marker of mesenchymal cells, was decreased in TMEM165KO cells compared to control. Cumulatively these results indicate a substantial phenotypic change in MDAMB231 cells following knockout of TMEM165.

### Loss of TMEM165 inhibits breast cancer cell migration and invasion

**Figure 3:**
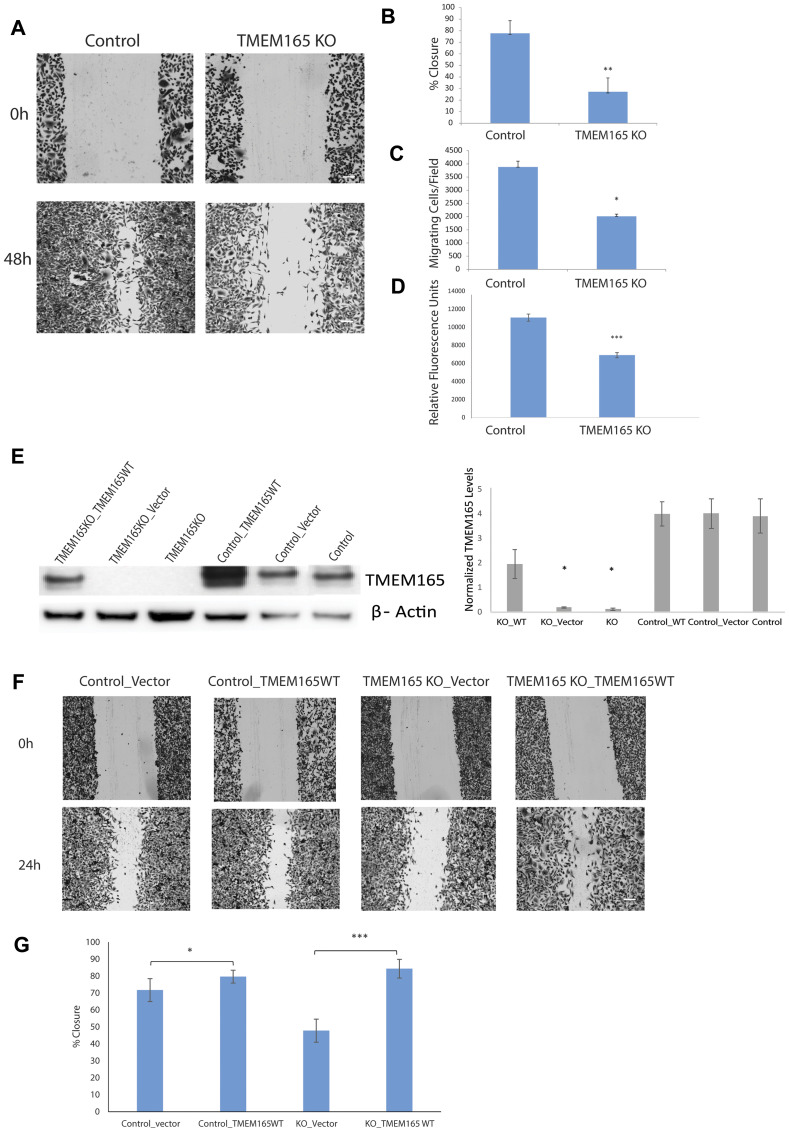
MDAMB231 TMEM165KO cells have reduced cell migration. (**A**) Representative phase-contrast images of wound healing assays captured at 0 and 48 hr time points. Bar = 300 μm (**B**) Graphical representation of the percent closure from *n* = 3 wound healing experiments analyzed using ImageJ. Error bars denote SD; ^**^ *P* < 0.001. (**C**) Boyden chamber transmigration data from *n* = 5 experiments showing the number of cells present on the lower compartment of a transwell membrane after 48 h. Error bars denote SD; ^*^ *P* < 0.05. (**D**) Chemicon (ECM554, Millipore, Billerica, MA) cell invasion assays were performed and cells that invaded ECM were measured using fluorescent plate reader, error bars denote SD of *n* = 3 experiments; ^***^ *P* < 0.001. (**E**) Western blot analysis showing TMEM165 expression after transient transfection with TMEM165/GFP plasmid and vector in control and TMEM165KO cells. Right panel shows the quantification of TMEM165 protein levels after normalization with actin (*n* = 2, ^*^ *P* < 0.05). (**F**) Representative phase-contrast images show migration rescue in TMEM165KO after transfection with TMEM165/GFP plasmid compared to the control at the 0 and 24 hr time points. Bar = 300 μm (**G**) Graphical representation of wound healing rescue experiments *n* = 3 analyzed using Image J. Error bars denote SD; ^***^ *P* < 0.000007 and ^*^ *P* < 0.05.

Our discovery of increased expression of TMEM165 in invasive breast cancer led us to investigate the potential role of TMEM165 in cell migration. We tested whether TMEM165 expression could alter cell migration and invasion induced by a wound healing assay or stimulated in a Boyden chamber transmigration assay. The analysis of wound healing cell migration assays showed an almost complete closure of the wound in control cells by 48 hrs post scratch with very limited closure of the wound in TMEM165KO cells (Figure 3A). Cumulative results from three separate experiments show highly significant reduction in cell migration (*p* < 0.001) Figure 3B. To further study the invasive potential of TMEM165KO cells, we performed Boyden chamber transmigration assays and found that MDAMB231 TMEM165KO cells have significantly (*p* < 0.05) reduced migration (Figure 3C). In addition to the Boyden transmigration assays we performed invasion assays to determine invasion though extracellular matrix. TMEM165KO cells show significant reduction in migration through extracellular matrix, Figure 3D (^***^ *P* < 0.0001). We have performed rescue experiments to confirm that TMEM165 expression is inducing the cell migration. Plasmids containing TMEM165 as a fusion with GFP [31] were transiently transfected into MDAMB231 TMEM165KO and control cell lines. Western blot analysis confirms that with no transfection or the transfection of vector only MDAMB231 TMEM165KO cells have no expression of TMEM165 (Figure 3E). The transfection of TMEM165/GFP fusion leads to increased expression of TMEM165 in the control cells with restored expression in TMEM165KO cells. Wounds healing migration assays were performed to demonstrate rescue of migration in MDAMB231KO cells after transfection with the TMEM165/GFP expression plasmid (Figure 3F and 3G, *P* < 0.000007). Furthermore, there is an increased migration in control cells following transfection with TMEM165/GFP (Figure 3G, *P* < 0.05). The rescue of migration in TMEM165/GFP transfected cells confirms a direct role for TMEM165 in the initiation of cell migration.

### Loss of TMEM165 inhibits breast cancer tumor growth *in vivo*

**Figure 4:**
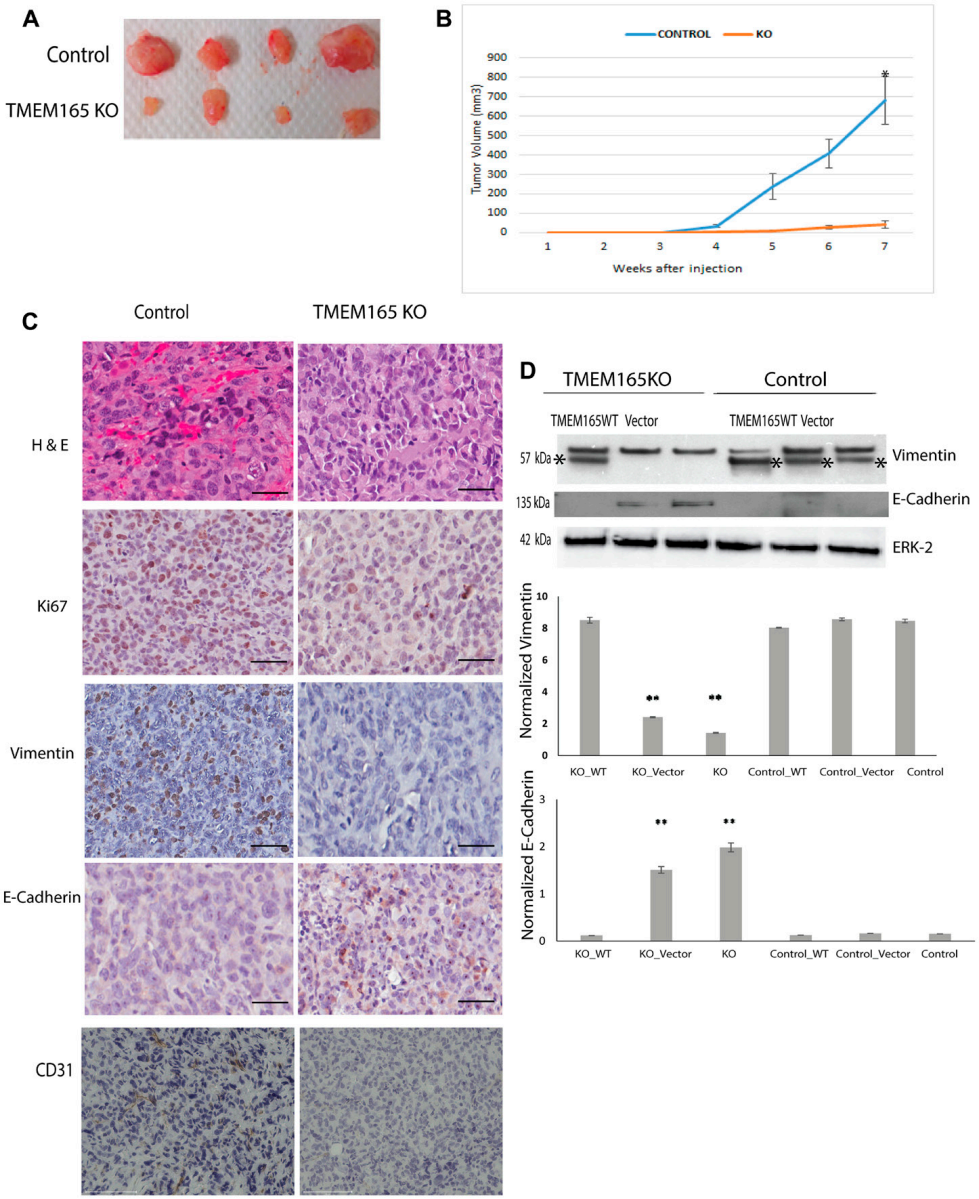
TMEM165 knockout inhibits breast cancer growth in a xenograft model. (**A**) Representative image of tumors extracted from nude mice treated with MDAMB231 Control and MDA MB231 TMEM165KO. (**B**) Cumulative growth curves for MDAMB231 control cells and MDAMB231 TMEM165KO cell xenografts (*n* = 10 mice control, *n* = 10 TMEM165KO; ^*^ *P* < 0.0001). (**C**) Representative images of MDAMB231 Control and TMEM165KO derived tumor sections stained for Hematoxylin and eosin (H&E), Ki67, Vimentin, E-Cadherin, and CD31, bars 100 μm. (**D**) Western blot analysis of vimentin and E-cadherin expression after transient transfection with TMEM165/GFP or vector in TMEM165KO and control cells, asterisks mark the vimentin band in Western blots. Lower panels shows the quantification of Vimentin and E-cadherin protein levels after normalization with ERK (*n* = 2, ^**^ *P* < 0.01). The Western blot images are cropped and were imaged with Bio-Rad EZ Imager. The second band from the left on the ERK Western blot has a slight air bubble just above the ERK band that does not impair quantitation.

Our *in vitro* data indicate that knockout of TMEM165 in MDAMB231 cells by CRISPR/Cas9 gene editing significantly reduces cell migration and increases cell spreading. These results led us to investigate the effect of TMEM165 expression on xenograft tumor growth *in vivo*. The tumors of mice injected with TMEM165KO cells showed a significant reduction in tumor growth and vascularization as compared to tumors from mice administered with control cells (Figure 4A representative tumors, Figure 4B cumulative for *n* = 10 mice each group). Immunohistochemistry analysis of xenograft tumors reveals a loss of blood vessels in the TMEM165KO tumors on the H & E staining (Figure 4C, top panel). Staining for the vascular endothelial cell marker CD31 (Figure 4C lower panel) revealed a reduction in TMEM165KO tumors confirming reduced tumor vascularization. Tumors from TMEM165KO cells have lower levels of the proliferation marker Ki67 compared with control tumors (Figure 4C). The TMEM165KO tumors have a decrease in vimentin and an increase in E-cadherin suggesting a reversion to a more epithelial phenotype compared to control tumors (Figure 4C). Rescue experiments with transfection of TMEM165/GFP into MDAMB231 TMEM165KO cells reduces E-cadherin levels and increases vimentin confirming that these changes are due to TMEM165 expression (Figure 4D). Cumulatively, these results indicate that TMEM165 promotes the growth and invasion of breast cancer.

### TMEM165 expression alters N-linked glycosylation

**Figure 5:**
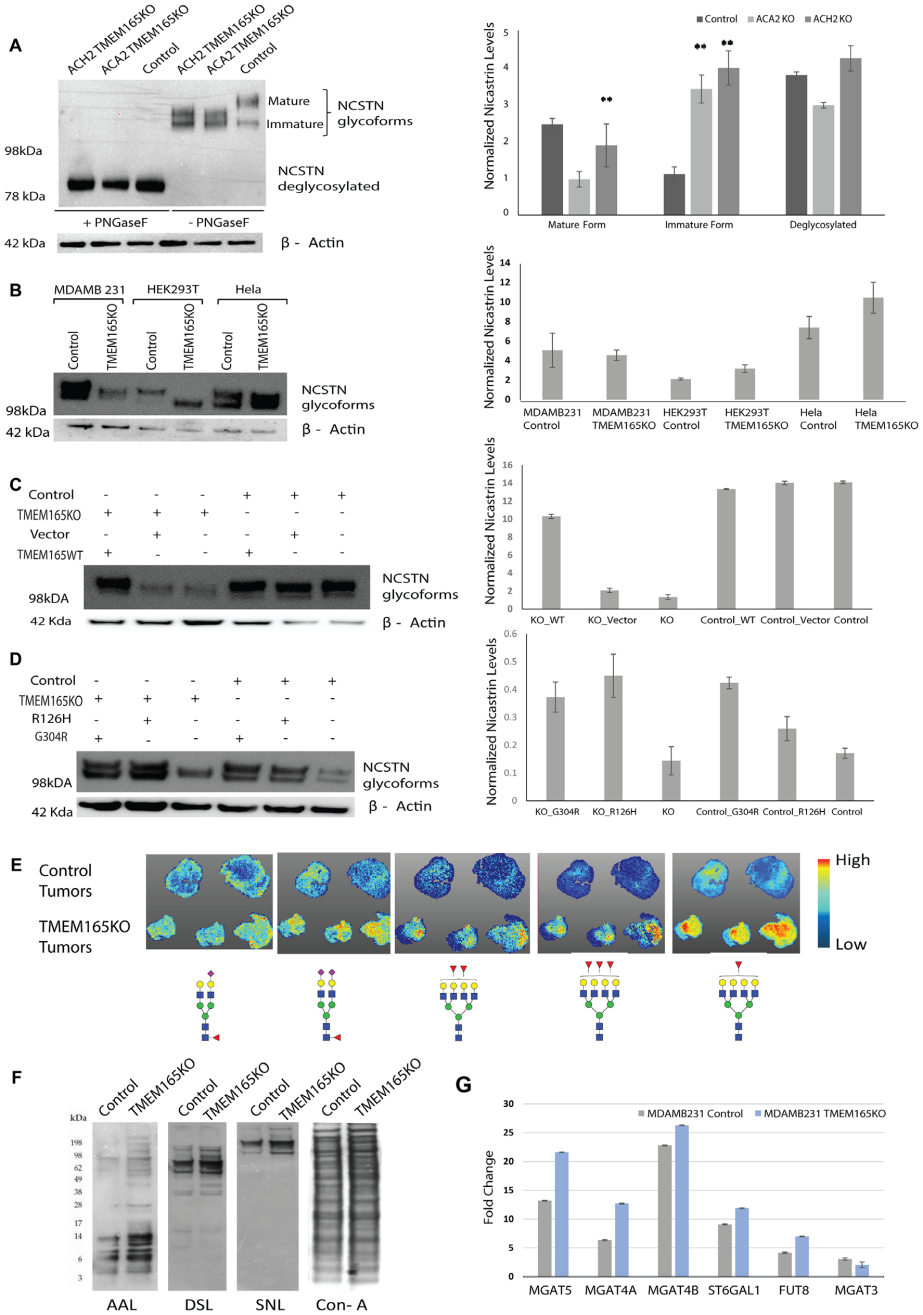
TMEM165 expression levels alters N-linked glycosylation. (**A**) Western blot analysis of NCSTN from MDAMB231 TMEM165KO (clones ACH2 and ACA2) and MDAMB231 Control (clone ACH11) cell lysates before and after PNGaseF treatment. NCSTN mature, immature, and deglycosylated bands are labeled. The Western blot was imaged on Bio-Rad EZ Imager and has not been manipulated. Any marks on the blot that are not labeled bands are from forceps handling of the membrane. Right panel shows the quantification of mature, immature and deglycosylated forms Nicastrin protein levels after normalization with actin (*n* = 2, ^**^ *P* < 0.01) (**B**) Western blot showing different glycoforms of NCSTN in Control and TMEM165KO across different cell lines (MDAMB231, HEK293T and Hela). NCSTN in Control and TMEM165KO across different cell lines (MDAMB231, HEK293T and Hela). Right panel shows the quantification of Nicastrin protein levels after normalization with actin (*n* = 2). (**C**, **D**) Western blot showing rescue of NCSTN glycoforms in TMEM165KO cells after transient transfection with TMEM16WT plasmid and TMEM165CDG mutants (R126H and G304R). Right panel shows the quantification of Nicastrin protein levels after normalization with actin (*n* = 2,^**^ *P* < 0.01). (**E**) Xenograft paraffin embedded tissue sections treated with PNGaseF were subjected to Tissue MALDI-FTICR IMS exhibited differential expression of N-glycan structures among Control and TMEM165KO tumor tissues. Represented glycan structures are in accordance with the guidelines for the Consortium for Functional Glycomics (CFG): blue square, N-acetylglucosamine (GlcNAc); green circle, mannose (Man); yellow circle, galactose (Gal); red triangle, fucose (Fuc); purple diamond, N-acetylneuraminic acid (Neu5Ac). (**F**) Lectin blot analysis of MDAMB231 control and TMEM165KO cell lysates. (**G**) Graph representing the relative transcript levels of MGAT5, MGAT4A, MGAT4B, ST6GAL1, FUT8 and MGAT3 in MDAMB231 control or TMEM165KO cells. Normalized data expressed as fold change are plotted for each gene assayed. Error bars represent one standard deviation from the mean of triplicate values from *n* = 3 experiments.

Mutations in TMEM165 are associated with congenital disorders of glycosylation. Patients with these mutations show distinct changes in the N-linked glycosylation of serum glycoproteins such as transferrin. Therefore, we want to evaluate whether TMEM165 alters N-linked glycosylation in breast cancer cells. MDAMB231 control and two different clones of TMEM165KO cells were subjected to PNGaseF treatment, which cleaves all N-linked glycans including high mannose, hybrid and complex structures at asparagine residues of N-linked glycoproteins. Both MDAMB231 TMEM165KO clones (ACH2 and ACA2) revealed dramatic differences in the migration of nicastrin (NCSTN) glycoforms. Nicastrin, an N-linked glycoprotein that is an essential component of γ-secretase, is required for proper Notch signaling [32]. The molecular weight of the NCSTN glycosylated variant in control MDAMB231 cells displays a higher molecular weight migration for the mature protein; while TMEM165KO clones display NCSTN glycoforms with a shift in migration of the mature protein toward a lower molecular weight (Figure 5A). Changes in the migration of glycoproteins can be due to changes in levels of charged glycans versus neutral glycans or may be due solely to truncation of glycan structures. NCSTN migration was the same in all cells following PNGaseF treatment indicating that the changes in migration are due to N-glycosylation on glycoproteins (Figure 5A). These results indicate that TMEM165 expression changes N-linked glycosylation in MDAMB231 cells.

The same CRISPR Cas9 guide RNA targets used in this study were previously used to create TMEM165KO in HEK293T and HeLa cell lines [14]. We evaluated NCSTN glycoforms in control and TMEM165KO for each of these cell lines and compared this to MDAMB231 cells. Our data show that TMEM165KO in HEK293T and HeLa cells display NCSTN glycoforms with migration to a lower molecular weight than the MDAMB231 breast cancer TMEM165KO cells (Figure 5B) These results indicate that the NCSTN glycoprotein from MDAMB231 breast cancer cells have different glycosylation compared with HEK293T and HeLa cells. To rescue the altered NCSTN glycoforms in TMEM165KO cells both TMEM165WT plasmid and empty vector were transiently transfected in MDAMB231 cells. These results further confirmed that altered glycosylation pattern observed in NCSTN was rescued only in TMEM165KO cells after transfected with TMEM165WT plasmid and not vector (Figure 5C). Interestingly, when CDG mutant forms of TMEM165 (R126H and G304R) were transiently expressed in MDAMB231 cells, N-linked glycosylation was also present with NCSTN glycoforms as observed in control cells (Figure 5D). These results indicate that the changes that occur in TMEM165 in CDG patients that disrupt function are not disrupting the function of TMEM165 in the MDAMB231 breast cancer cells as these mutants were capable of inducing the glycoform of nicastrin observed in MDAMB231 control cells.

We sent xenograft tumors from control and TMEM165KO for analysis using Maldi FTICR IMS N-glycan imaging to get a preliminary evaluation of glycosylation variants (Figure 5E). In this method FFPE tissue sections are treated with PNGaseF to release the N-linked glycans from glycoproteins. This method allowed us to spatially localize individual glycan structures to specific tissue regions [33]. A total of 83 N-glycans were detected. Many N-linked glycan structures did not show a change in abundance between control and TMEM165KO (data not shown). However, certain structures showed differences in abundance. Overall, our results demonstrate an increase in bi-antennary sialylated glycans in TMEM165KO tumors with fucose and an increase in tetra-antennary glycoforms with core and/or outer fucosylation (Figure 5E). We confirmed these findings using analysis of the MDAMB231 control and TMEM165KO cells using lectin blot analysis and qRT-PCR analysis. The lectins AAL, DSL, and SNA recognize core and outer fucose, β1,4 branched glycans [34, 35], and sialic acid linked to terminal galactose, respectively [36]. Our lectin blot analysis confirms an increase in the levels of these N-linked glycan structures on very specific glycoproteins in TMEM165KO cells (Figure 5F). There are distinct glycoproteins with different molecular weights showing changes in lectin binding. For example, fucose changes detected by AAL are distributed in low and high molecular weight glycoproteins. However, changes in sialylation detected by SNA were limited to high molecular weight glycoproteins. We performed a lectin blot with ConA to demonstrate an equivalent loading of glycoproteins in each lane. Glycan levels are often changing as a result of changes in the levels of glycosyltransferase (GT) enzymes. We measured the levels of certain GT enzymes that produce the N-glycan structures that were increased in TMEM165KO xenograft tumors using real-time PCR analysis. Data shown in Figure 5G show the fold change in relative normalized expression levels for each enzyme in control and TMEM165KO cells. The changes in GT mRNA levels correlate well with the lectin blot data and the Maldi-FTICR IMS imaging data suggesting that TMEM165 expression levels change the transcript levels of these GT enzymes in the N-linked pathway.

## DISCUSSION

EMT is an essential component of normal development that is involved in tissue regeneration and cancer cell invasion [21]. EMT is a dynamic process and studies to identify key regulators will allow for novel methods to control this pathway for tissue regeneration and prevention of cancer invasion in the future. Mutations in the TMEM165 gene have been linked to congenital diseases of glycosylation where the patients have significant bone and cartilage growth defects [12, 13]. Therefore, TMEM165 may be a protein required for mesenchymal transitions. We discovered TMEM165 as a biomarker for IDC in a glycoproteomics analysis using high grade IDC tissues with matched adjacent normal [10]. The current study has extended our previous discovery and demonstrates that TMEM165 induces glycosylation changes and protein expression changes in breast cancer that favors EMT. We are the first to report that increased expression levels of TMEM165 in IDC tumors correlates with poor prognosis in breast cancer patients. Additionally, we have shown that TMEM165 is expressed in DCIS and increases in early stage IDC making it a potential driver of tissue invasion. This study is also the first to use CRISPR/Cas9 gene editing to delete TMEM165 in a human breast cancer cell line. Our xenograft results confirm that TMEM165 expression promotes the expression of proteins such as vimentin and suppress E-cadherin expression promoting the shift to a more mesenchymal phenotype. These findings place TMEM165 as a regulator for the epithelial to mesenchymal transition.

In CDG patients, TMEM165 mutations have been shown to lead to changes in protein splicing that disrupt the function of TMEM165 leading to glycosylation changes [12, 31]. In breast cancer, we find that TMEM165 is amplified in expression with no identified mutations. A previous study observed increased expression of TMEM165 in hepatocellular carcinoma (HCC) and knockdown of TMEM165 in HCC led to a decrease in invasive disease [37]. We find that TMEM165 expression increases in IDC and patients with higher TMEM165 expression levels have a significant reduction in overall survival. This places TMEM165 as a potential target for the development of new therapeutic strategies to improve patient survival.

The functional significance of how TMEM165 alters N-linked glycosylation is not well understood and the changes in glycosylation in human patients and zebrafish models are variable [16, 38, 39]. Possible explanations include that TMEM165, a Golgi transmembrane protein and putative ion transporter, is acting as a Mn2+/Ca2+ transporter moving Mn2+ from the Golgi lumen to the cytosol. Evidence supports a role for TMEM165 in Mn++ transport as the TMEM165 protein is degraded by high cytosolic Mn2+ concentration [14, 40]. In breast cancer, calcium ATPases are altered in expression levels and activity and these findings would further complicate the control of ion homeostasis [41, 42]. This may account for our observation that changes in N-linked glycans in the MDAMB231 breast cancer cells with TMEM165KO yields a very different migration pattern of the glycoprotein NCSTN compared with the non-malignant HEK293T cells and the cervical cancer cell line HeLa. Our Maldi-FTICR-IMS imaging results indicate an increase in sialylation and fucosylation on specific N-linked structures in our xenograft TMEM165KO tissues compared to control tissues. Our previous glycomic analysis of IDC patient tissues revealed an increase in tetra-antennary fully sialylated structures with polylactosamine in tumor tissue compared to patient matched normal [43]. In this study we find that knockout of TMEM165 expression is changing the expression of key glycosyltransferases such as Mgat5, Mgat4a, FUT8, and ST6GAL1. Each of the GT enzymes changing in TMEM165KO tissues has been linked to promoting cancer invasion in previous studies [44–48]. However, our xenograft data clearly indicate that there is a significant reduction in invasion, vascularization, and growth in TMEM165KO tumors despite the increase in N-glycan structures thought to promote invasion. Our current data indicate there may be more functional significance of the specific N-linked glycan structures presented at asparagine sites located on certain glycoproteins that is important for influencing tumor invasion, as indicated in previous studies examining changes in glycosylation in clinically advanced breast cancers [49, 50]. Alternatively, TMEM165 may be leading to changes in the expression levels of glycoproteins that function to shift toward a more invasive phenotype and this global glycoprotein change drives the phenotype changes. This theory would agree with the fact that variable changes in N-glycan processing have been observed in serum proteins from human CDG patients and these patients all share significant skeletal and cartilage defects [12, 39, 51]. Future mass-spectrometry studies identifying the glycoproteins that change expression in response to TMEM165 expression are needed to better understand the influence of TMEM165 on cell phenotype.

Our data indicates that both wild-type (WT) TMEM165 and CDG mutant forms of TMEM165 can change the glycosylation of NCSTN in MDAMB231 TMEM165KO cells back toward the migration observed in control cells. We also find that WT and CDG Mutant forms of TMEM165 can both rescue cell migration in the TMEM165KO cells (data not shown). These findings indicate that the mutations in CDG patients associated with disease defects and changes in glycosylation for these patients during early development are distinct from the regions of the TMEM165 protein controlling migration, glycosylation changes, and phenotype in breast cancer.

Our analysis of TMEM165 expression levels in human breast cancer cell lines suggests that there may be a relationship between ER expression and the control of TMEM165 expression levels. Human breast cancer cell lines with ER expression have undetectable levels of TMEM165 by RT-PCR and Western blot; while cell lines without ER expression have higher levels of TMEM165. Our TCGA expression analysis indicates that certain ER+ breast cancers do express increased TMEM165 (Table 2) although the overall percentages of luminal A and luminal B cases with TMEM165 expression are lower than breast cancers that are negative for ER receptor. We have examined the levels of TMEM165 in ER- and ER+ metastatic TCGA breast cancer cases and find that there are significantly higher TMEM165 levels in ER- metastatic cases (*p* 0.009, Supplementary Figure 2). These findings support further study as many women with ER + breast cancers are treated with ER antagonist. It will be important to determine if the TMEM165 gene is regulated by the ER receptor and how the levels of TMEM165 change in response to ER receptor antagonist such as tamoxifen. For example, would ER antagonist treatment lead to an increase in TMEM165 over time and could this be linked to recurrence of invasive disease? TMEM165 has recently been linked to milk production during lactation and these findings would also support potential hormonal regulation of TMEM165 [19, 20].

In conclusion, we have expanded on our initial 2012 glycoproteomic study that was the first to identify TMEM165 protein as a potential biomarker for breast carcinoma. In this study we have provided initial mechanistic studies that indicate that TMEM165 expression drives the growth and invasion of breast cancer. TMEM165 expression levels could be a potential prognostic marker for predicting DCIS cases that may progress to invasive disease. Larger prospective cohorts will need to be analyzed to determine the link between TMEM165 levels and the progression to IDC. We find that IDC patients with higher TMEM165 expression levels have reduced overall survival making this protein a target for the development of new therapeutic strategies to limit the progression of breast cancer.

## MATERIALS AND METHODS

### Kaplan–Meier plot analysis

The prognostic value of the *TMEM165* gene in breast cancer was analyzed using the online Kaplan–Meier plotter (http://kmplot.com/analysis/) [52–54]. For analysis, patients with breast cancer were divided into two groups according to higher and lower *TMEM165* expression levels (specifications regarding cutoffs are included in the figure legend). The overall survival (OS) was then compared between the higher and lower expression groups. Log rank *p* values and hazard ratios with 95% confidence intervals were then calculated.

### Gene expression analysis via UALCAN

UALCAN (http://ualcan.path.uab.edu/) is an online, open-access platform for visualization of gene expression alterations occurring between cancerous and paired normal tissues relative to clinical pathological features, which are sourced from the TCGA database [18]. In the current study, UALCAN was applied to analyze the transcriptional levels of TMEM165 in primary breast cancer tissues and its association with molecular subtypes. All the breast cases available on UALCAN were included in our research.

### Scratch wound assay

MDAMB231 cells were seeded into a 6-well plate at high density in DMEM (Invitrogen) with 10% FBS (Gemini Bioproducts, West Sacramento, CA) and 100 mg/mL streptomycin (Invitrogen). A clear area was scraped into the cell monolayer using a pipet tip. Cells were washed gently with PBS to remove the detached cells, placed in complete growth media for 2 days. Migration of cells into the wounded area was monitored by microscopy using a Cytation 3 (BioTek) and images were captured at different time points after the scratch (0 hr, 24 hr and 48 hr). Wound closure was quantified using ImageJ software (http://imagej.nih.gov/ij/docs/index.html).

### Boyden chamber assay

Boyden chamber assay was preformed using Cell culture inserts Transparent PET membrane (Corning, Life sciences, USA) with 8 μm pore size in 24-well dishes. Approximately 4 × 10^5^ cells in 200 μl of complete medium were seeded into the upper chamber and 500 μl of complete medium placed in the lower chamber. The next day, the cells in the upper chamber were replenished with serum-free medium. The lower chamber was replaced with complete medium to allow migration of cells for 48 hrs. Cells on the inside of the filters were removed and cells that had migrated to the underside of the filters were stained with crystal violet. Five random fields from *n* = 3 experiments were counted.

### Invasion assay

Chemicon invasion assays (ECM554, Millipore, Billerica, MA) were used to test invasiveness. Briefly, cells were washed in serum-free medium, a cell suspension made at 0.5 × 10^6^ cells/ml. 250 μL cell suspension was then added to rehydrated invasion membranes. Serum (10%) in serum-free medium was added to the lower chamber as chemoattractant and the plates incubated for 24 hrs at 37°C before detection, according to manufacturer’s instructions using a fluorescent plate reader.

### Cell staining

MDAMB231 control and MDAMB231 TMEM165KO cells were plated on coverslips that were pre-coated with 10 μg/mL Fibronectin. Cells were allowed to adhere overnight in 37°C CO2 incubator. Attached cells were washed twice with PBS then fixed with 4% Paraformaldehyde for 20 minutes and washed twice with PBS. Cells were permeabilized with 0.1% Triton X-100 in PBS for 15 min, washed with PBS, then stained with Alexa Fluor 568-conjugated phalloidin 1:40 dilution (Invitrogen, Molecular Probes). Cells for staining with vimentin and E-cadherin were fixed in cold methanol for 5 minutes prior to blocking and addition of primary antibodies against Vimentin and E-Cadherin (1:200 dilution) (Cell Signaling) in PBS-T/1% BSA overnight at 4°C. The next day after washing the cells were incubated with Alexa Fluor 488 conjugated goat anti-rabbit for 1 hr at room temperature. Nuclei were counterstained with a 1:10,000 solution of DAPI for 10 seconds before mounting in Prolong Gold Antifade (ThermoFisher). Stained cells were imaged using EVOS M7000 microscope.

### Western blot

Cells were harvested in RIPA buffer (1× PBS, 1% Nonidet P-40, 0.5% sodium deoxycholate, 0.1% SDS. BT549 and HS57T cell lysate were purchased from Origene. 20 μg total lysate protein was heated to 60°C for 5 mins in SDS sample buffer and separated on 4–12% Bis Tris polyacrylamide gels (Invitrogen) before transfer to PVDF membrane. Membranes were blocked overnight at 4°C in 5% nonfat milk/1XTBS/.05% tween-20 (TBST Blotto). After blocking the membranes were incubated in primary antibodies anti-TMEM165 (1:1000) and anti-β Actin (1:500) antibodies (Sigma–Aldrich), anti-E-caderin (1:500) (Cell Signaling), anti-vimentin 1:1,000 (Cell Signaling) anti –Nicastrin (1:500) (R&D Systems) diluted with 1× TBST Blotto for 1 hr at room temperature. After washing, the membranes were incubated with goat anti-rabbit or goat anti-mouse immunoglobulins HRP conjugated were purchased from Santa Cruz diluted (1: 10,000) in TBST at room temperature for 30 min. Washed blots were developed in ECL substrate (Thermo Scientific).

### Mouse xenograft model

All procedures were performed at The University of Arkansas for Medical Sciences (UAMS) under an approved animal use protocol. All methods were performed in accordance with guidelines and regulations at UAMS. UAMS maintains a centralized animal care and use program accredited by the Association for Assessment and Accreditation of Laboratory Animal Care International (AAALAC) and has an assurance on file with the Office of Laboratory Animal Welfare (OLAW). Six-week-old female immunodeficient mice were obtained by Jackson Labs NSG, NOD *scid*/6 weeks old. Two million cells, MDAMB231 Control or TMEM165KO, were injected subcutaneously into the right or left flank (respectively) of *n* = 10 mice. Mice were monitored daily for 6 weeks prior to necropsy and tumor extraction. Tumor volume in mg is calculated using the following formula: volume _ (length × width^2^/2).

Procedures were performed at The University of Oklahoma Health Sciences Center under an approved animal use protocol. All methods were performed in accordance with guidelines and regulations at OUHSC. Mouse mammary gland with human DCIS xenograft tissue sections were obtained from Dr. Bethany Hannafon. NOD *scid* gamma mice were injected with 10,000 MCF10DCIS. com cells per gland (Left and Right) and allowed to develop into DCIS and IBC. The mice were sacrificed after 40 days and tumor sections were collected.

### Immunohistochemistry staining

Paraffin-embedded tissue sections from control and TMEM165KO tumors were de-waxed and re-hydrated. Tissues were blocked with 0.5% hydrogen peroxide for 30 minutes. Tissues were rinsed in PBS with 0.2% tween 20 prior to blocking in 2.5% blocking serum (Vector Labs) for 20 minutes. The antibodies Ki67 (Santa Cruz Biotechnology) (1:100), E-cadherin (Cell Signaling) (1:200), vimentin (Cell Signaling) (1:200), or CD31 (Abcam) (1:50) were diluted in PBS/0.2% tween 20 and added for 1 hr at room temperature. Following washes in PBS/0.2% tween 20 the tissues were incubated with the ABC reagent (Vector Labs) for 30 minutes. Positive staining was detected using DAB substrate followed by hematoxylin counterstain and mounted. Images were captured with an Aperio Scanscope (Leica Biosystems).

### CRISPR-Cas9 gene editing

MDAMB231 cells were maintained in DMEM media supplemented with 10% FBS without antibiotics. For creation of the CRISPR KO cells, media was changed to 2 mL OPTI-MEM without FBS/antibiotics and transfected using Life Technologies recommended protocol for MDA-MB-231 cells (Lipofectamine 3000 with Plus reagent). Transfections were carried out in a 6-well plate using an equal amount of Cas9 DNA plus TMEM165 CRISPR “A” guide RNA target sequence (GeneCopoeia) totaling 2.5 ug of DNA per well. Transfection was allowed to be carried out for 5 hrs at which time the transfected cells were supplemented with an equal volume of standard media. Twenty four hrs after transfection the media was completely changed to standard media. Two weeks after transfection, live cells were single cell sorted into 3 × 96 well plates. Single cell colonies were grown to at least 50% confluence and split into 24-well plates to be tested by immunofluorescence for staining with TMEM165 antibodies. Colonies that did not stain with TMEM165 antibodies were validated as KOs by Western blot and DNA analysis.

### Glycosidase digestion

To remove *N-*glycans, protein extracts were digested with PNGaseF using the protocol as described by the vendor New England Biolabs. Briefly, 20 μg of glycoprotein were combined with 1 μl of Glycoprotein Denaturing Buffer (10×) and H_2_O to make a 10 μl total reaction volume. To denature glycoproteins, the reaction was subjected to heat at 100°C for 10 minutes. The denatured glycoproteins were chilled on ice and centrifuged 10 seconds. To make a total reaction volume of 20 μl, 2 μl GlycoBuffer 2 (10×), 2 μl 10% NP-40 and 6 μl H_2_O was added. 1 μl PNGase F was added and gently mixed. The reaction was incubated at 37°C for 1 hr. Then the samples were run on SDS- polyacrylamide gel to analyze the extent of de-glycosylation.

### Lectin blot analysis

The cells were lysed in RIPA buffer (1× PBS, 1% Nonidet P-40, 0.5% sodium deoxycholate, 0.1% SDS) containing protease inhibitor. The lysate was cleared by centrifugation at 10 000× g for 10 min. Protein concentrations were determined by BCA assay (Pierce, Rockford, IL). Twenty μg of proteins were separated on 4–12% NuPage Bis Tris gels and transferred to PVDF membrane at 25 V for 1.5 h. Membranes were blocked overnight in 3% BSA/TBST buffer before lectin blot detection using a 1:5000 dilution of the following biotinylated lectins: (Sambucus nigra (SNL), (SNL), Aleuria aurantia (AAL), and Datura stramonium (DSL), Concanavalin A (ConA) (Vector Labs). Bound lectin was detected using a 1:10,000 dilution of streptavidin–HRP (Vector Labs) before washing and detection using Western Lightening Plus (Perkin Elmer).

### Real time PCR

qRT-PCR was performed as described previously [55]. Briefly, Samples (50 μl of packed cells) were extracted using TRIzol (Invitrogen) according to the manufacturer’s instructions. After DNase treatment, RNA (2 μg) was reverse-transcribed using Superscript III (Invitrogen) with random hexamers and oligo (dT). Primers were validated with respect to primer efficiency and single product detection. The primer sequences are shown in Supplementary Table 1. Quantitative real-time PCR was performed in a Bio-Rad CFX96 C1000 instrument using RT SYBR Green/Fluorescein qPCR Master Mix (Bio-Rad). Relative expression of target genes was calculated using the comparative cycle threshold method with genes normalized to RPL4. Triplicate Ct values for each gene were averaged and the SD from the mean was calculated.

### Tissue MALDI imaging

Tissue MALDI imaging was performed as described previously using a standardized protocol [33, 50, 56]. Briefly, formalin-fixed paraffin embedded tumor tissues were antigen retrieved followed by spraying of a molecular coating of PNGaseF using a TMSprayer (HTX Technologies LLC., Chapel Hill, NC). After a 2 hr incubation, slides were dessicated and 7 mg/ml CHCA matrix was applied using the TMSprayer. Slides were stored in a desiccator until MALDI-FTICR MS analysis. Spectra were acquired across the entire tissue section on a Solarix dual source 7T FTICR mass spectrometer (Bruker Daltonics) to detect the N-glycans with a SmartBeam II laser operating at 2000 Hz, a laser spot size of 25 μm and a raster width of 125 μm unless otherwise indicated. Images of differentially expressed glycans were generated to view the expression pattern of each analyte of interest using FlexImaging 4.0 software (Bruker Daltonics) and structural representations of these m/z values were generated using GlycoWorkbench based on previous studies [57].

### Transfection

MDAMB231 Control and TMEM165KO cells were transfected with TMEM165/GFP WT, TMEM165/GFP R126H, TMEM165/GFP G304R or vector plasmid. Transfections were performed using lipofectamine 2000 (Invitrogen) following the instructions of the manufacturer. After 24 hrs of transfection, scratch wound assay was performed as described above and cells were scraped for western blot analysis.

## SUPPLEMENTARY MATERIALS



## Abbreviations

CRISPR: clustered regularly interspaced short palindromic repeats
Cas9: CRISPR associated protein 9
TMEM165: transmembrane protein 165
EMT: epithelial-to-mesenchymal transition
IDC: invasive ductal carcinoma
DCIS: ductal carcinoma *in situ*
NCSTN: nicastrin
CDG: congenital diseases of glycosylation
FTICR: Fourier-transform ion cyclotron resonance
AAL: Aleuria Aurantia Lectin
DSL: Datura Stramonium Lectin
SNA: Sambucus Nigra Lectin
