# Supplementary material for: Novel role for the Golgi membrane protein TMEM165 in control of migration and invasion for breast carcinoma
Source: Oncotarget. 2020 Jul 14;11(28):2747–62. doi: 10.18632/oncotarget.27668 (PMC7367651; doi:10.18632/oncotarget.27668)
Supplement: Supplementary file 1 [file oncotarget-11-2747-s001.pdf]

Novel role for the Golgi membrane protein TMEM165 in control of migration and invasion for breast carcinoma

SUPPLEMENTARY MATERIALS

The University of California Santa Cruz (UCSC) Xena browser (http://xena.ucsc.edu/), an analytics, visualization and integration tool for analyzing and viewing public data hubs, was adopted to assess *TMEM165* expression in relation to ER status as sourced from the TCGA breast cancer data [1]. For gene expression analysis, RNA-Seq (polyA+ Illumina HiSeq) data was downloaded as log2 (norm_count + 1) values.

REFERENCES

1. Goldman M, Craft B, Hastie M, Repečka K, McDade F, Kamath A, Banerjee A, Luo Y, Rogers D, Brooks AN, Zhu J, Haussler D. The UCSC Xena platform for public and private cancer genomics data visualization and interpretation. bioRxiv. 2019 Mar 5. [Epub ahead of print]. https://doi.org/10.1101/326470.

Supplementary Table 1: Real-time PCR primer sequences

Gene	Gene Code	Species	Accesssion	Forward Primer (5′ to 3′)	Reverse Primer (5′ to 3′)
Mannoside acetylglucosaminyltransferase 3	MGAT3	Human	NM_002409	CGAGGGCATCTACTTCAAGC	CGCTTGTCCTCGTAGTCACC
Mannoside acetylglucosaminyltransferase 4, isoenzyme A	MGAT4a	Human	NM_012214	AAGGTCTACCAAGGGCATACG	TATCGGTGTGATAGCCCAGAA
Mannoside acetylglucosaminyltransferase 4, isoenzyme B	MGAT4b	Human	NM_014275	TGCACTCGTACCTGACTGACA	GACCGAGTCCTCCTTCTCCT
Mannoside N-acetylglucosaminyltransferase V	MGAT5	Human	NM_002410	AGCCTGAAAGCAGCTCCAT	GCCAGTGCCTTGATGTACCT
Fucosyltransferase 8	FUT8	Human	NM_178154	GGTCGAGCTTCCCATTGTAG	GCGAGGTCTTCTGGTACAGC
Beta-galactoside alpha-2,6-sialyltransferase 1	ST6GAL1	Human	NM_173216<br>NM_003032<br>NM_173217	TATCTGCCCAAGGAGAGCAT	GACGACACAACAGCACACCT
Ribosomal Protein L4*	RPL4	Human	NM_000968	AGAAGGCTGCTGTTGGTGTT	TGGTTTCTTGGTAGCTGCTG

*Normalization control gene.

A

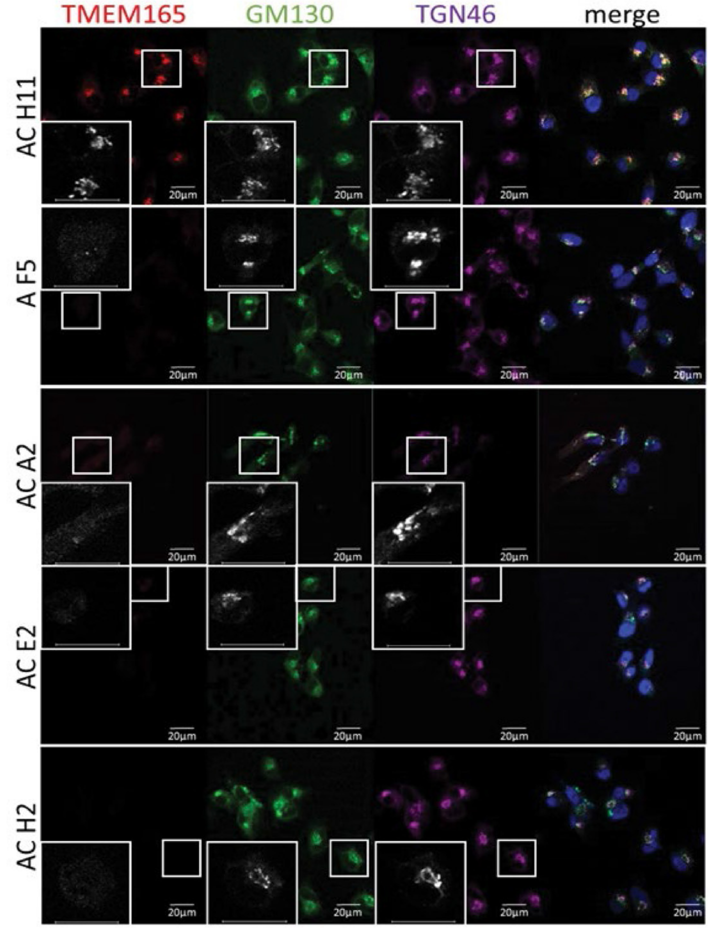

B

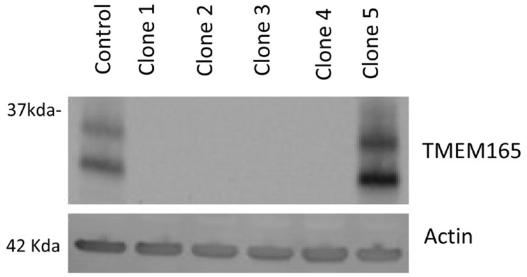

MDA Clone ID
Clone 1: A F5
Clone 2: AC A2
Clone 3: AC E2
Clone 4: AC H2
Clone 5: AC H11

Supplementary Figure 1: (**A**) Immunofluorescence (IF) staining of TMEM165 and Golgi markers in MDA MB 231 CRISPR/Cas9 clones. Clone AC H11 still contains TMEM165, while Clone A F5, AC A2, AC E2, and AC H2 do not contain any detectable TMEM165 by IF. (**B**) MDA MB 231 TMEM165 clones tested by Western blot for TMEM165 expression.

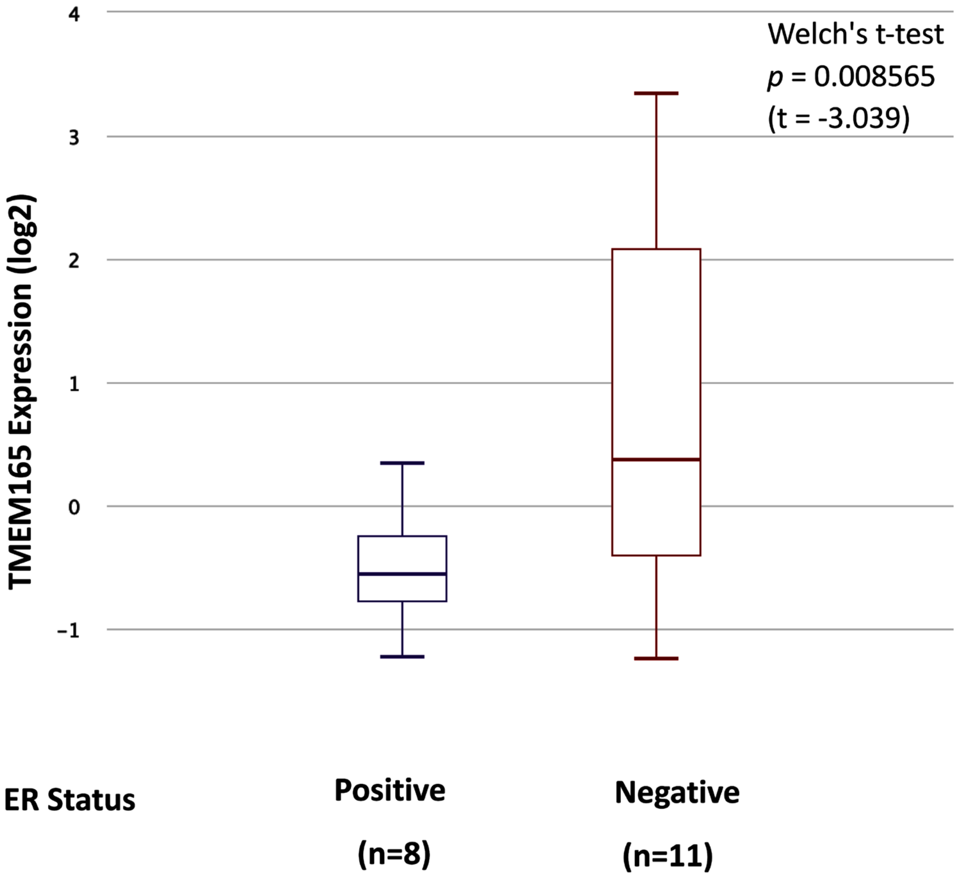

Supplementary Figure 2: *TMEM165* expression varies significantly with regard to ER status of breast cancer. Box plots of TMEM165 expression relative to ER status of breast cancer (TCGA Database). Significance between the groups was assessed by Welch's *t*-test.
